# Age-dependent Changes in the Articular Cartilage and Subchondral Bone of C57BL/6 Mice after Surgical Destabilization of Medial Meniscus

**DOI:** 10.1038/srep42294

**Published:** 2017-02-09

**Authors:** Henry Huang, Jordan D. Skelly, David C. Ayers, Jie Song

**Affiliations:** 1 University of Massachusetts Medical School, Department of Orthopedics & Physical Rehabilitation, Worcester, MA 01655, USA

## Abstract

Age is the primary risk factor for osteoarthritis (OA), yet surgical OA mouse models such as destabilization of the medial meniscus (DMM) used for evaluating disease-modifying OA targets are frequently performed on young adult mice only. This study investigates how age affects cartilage and subchondral bone changes in mouse joints following DMM. DMM was performed on male C57BL/6 mice at 4 months (4 M), 12 months (12 M) and 19+ months (19 M+) and on females at 12 M and 18 M+. Two months after surgery, operated and unoperated contralateral knees were harvested and evaluated using cartilage histology scores and μCT quantification of subchondral bone plate thickness and osteophyte formation. The 12 M and 19 M+ male mice developed more cartilage erosions and thicker subchondral bone plates after DMM than 4 M males. The size of osteophytes trended up with age, while the bone volume fraction was significantly higher in the 19 M+ group. Furthermore, 12 M females developed milder OA than males as indicated by less cartilage degradation, less subchondral bone plate sclerosis and smaller osteophytes. Our results reveal distinct age/gender-dependent structural changes in joint cartilage and subchondral bone post-DMM, facilitating more thoughtful selection of murine age/gender when using this surgical technique for translational OA research.

Age is the primary risk factor for developing osteoarthritis (OA), a chronic, degenerative joint disease that is characterized by progressive loss of articular cartilage and subchondral bone changes. While joint space narrowing, subchondral bone sclerosis and osteophyte formation are all clinical signs of OA, the temporal nature of these changes and how they contribute to OA etiology are still an ongoing debate^1^,^2^. More recently, the notion that articular cartilage and subchondral bone should be viewed as a single unit due to their proximity and evidence of biomechanical and molecular signaling crosstalk has gained traction^3^,^4^,^5^. Osteophytes have also been regarded as a compensatory response to mechanical weakening of the degrading cartilage^6^, but existing evidence remains limited. Therefore, understanding how bone and cartilage structures change during OA progression as a function of age is essential to better understand OA pathology, elucidate key cellular and molecular players, as well as to develop disease modifying drugs.

Murine OA models are invaluable tools for both basic and translational research due to the ease of manipulating mouse genome. OA can be induced in murine articular joints by collagenase injections^7^, surgical destabilization^8^, or non-invasive injury techniques^9^. Surgical destabilization of the medial meniscus (DMM) results in gradual deterioration of articular cartilage and subchondral bone changes, and is thought to recapitulate the progression of human OA pathology^10^,^11^. Although DMM emulates post-traumatic OA by nature, murine DMM models have been broadly used by many studies to identify disease-modifying OA targets such as FGF1^12^, progranulin^13^, IGF-II^14^, DKK1^15^, DDR2^16^, HIf2alpha^17^ and ADAMTS5^18^. Recently, our lab has shown that Smurf2-deficiency mitigates cartilage degradation following DMM surgery, however, this benefit is reduced with advanced age^19^. In order to elucidate how age impacts the efficacy of various therapies to DMM-induced OA, it is critical to understand what age-dependent changes occur in articular cartilage and subchondral bone after joint destabilization.

Most DMM-induced OA studies perform surgery on 2–3 month old male mice and analyze their OA phenotypes 1–2 months later. While 2 month old mice are considered skeletally mature adults, their skeletal tissues continue to undergo significant changes well beyond 2 months. For instance, C57BL/6 mice femurs do not reach their maximum length until 6 months of age, and osteoporotic changes in the trabecular bones are not apparent until 6–12 month in females and over 12 months in males^20^. Aged mice also undergo significant weight changes, where it almost doubles by 12 months due to body fat accumulation^20^. Such age-related skeleton and body mass changes may reflect more accurately the biochemical and mechanical environment of human OA joints of middle-aged and older populations. Understanding how aged mice respond to DMM differently can enhance the translatability of this murine model to human OA. In addition, young (~4 M) female mice were found to be less prone to developing OA following DMM compared to their male counterparts^21^, but whether the perceived protective effect of sex hormone persists over age is unclear.

While prior human studies^22^,^23^,^24^ have established correlations among articular cartilage erosions, subchondral bone sclerosis and osteophyte formation, whether and how age impacts such structures in the mouse DMM OA model has not been carefully investigated. Previous studies that evaluated the effects of age on DMM-induced OA only examined young mice with 1 month age difference prior to DMM^25^ or focused their study on differences in gene expressions^26^. This study aims to understand how different tissue compartments in the knees of wild-type C57BL/6 mice from different age groups respond to DMM surgery by using cartilage histology scoring and quantitative micro-computed tomography (μCT) analyses of subchondral bone plate thickness and osteophyte formation. In addition, we also evaluate whether female hormones continue to provide protective effect on DMM-induced OA by comparing joint phenotype differences between middle-aged males and females at 2 months post-DMM.

## Results

### Severity of cartilage loss after DMM surgery was exacerbated in older male mice

**Figure 1:**
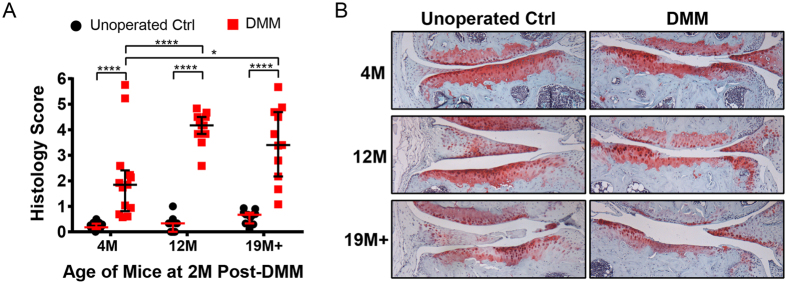
(**A**) Histological score of the tibial articular cartilage of unoperated and DMM operated knees of male mice from different age groups. Individual scores are plotted along with median score and interquartile range. *p-value < 0.05, ****p-value < 0.0001; (**B**) Representative safranin-o/fast green stained histological sections of the articular cartilage of unoperated and DMM operated knees of male mice from different age groups.

Cartilage histological scores of the medial tibiae between DMM-operated and unoperated contralateral knees of male mice from three different age groups revealed that the severity of OA cartilage 2 months post-DMM was significantly higher in the 12 M and 19 M+ group than in the 4 M group (Fig. 1A,B). The median histological score of unoperated knees for the 4 M, 12 M and 19 M+ groups was 0.18 (0.11–0.33), 0.33 (0–0.33) and 0.67 (0.33–0.75), respectively, consistent with very mild, non-structural spontaneous OA cartilage changes with age. The median histological score of the DMM knees in the 4 M, 12 M and 19 M+ groups was 1.8 (0.81–2.4), 4.2 (3.8–4.5) and 3.4 (2.2–4.7), respectively. Representative images of the articular cartilage in unoperated contralateral knees showed only progressive loss of safranin-O/fast green staining but minimal structural change with age, with superficial fibrillations only observed in some of the 19 M+ group. By contrast, the articular cartilage of DMM-operated knees showed increasing severity of cartilage erosion beyond the tidemark with age (Fig. 1B).

### Subchondral bone plate thickening after DMM surgery was greater in older male mice

**Figure 2:**
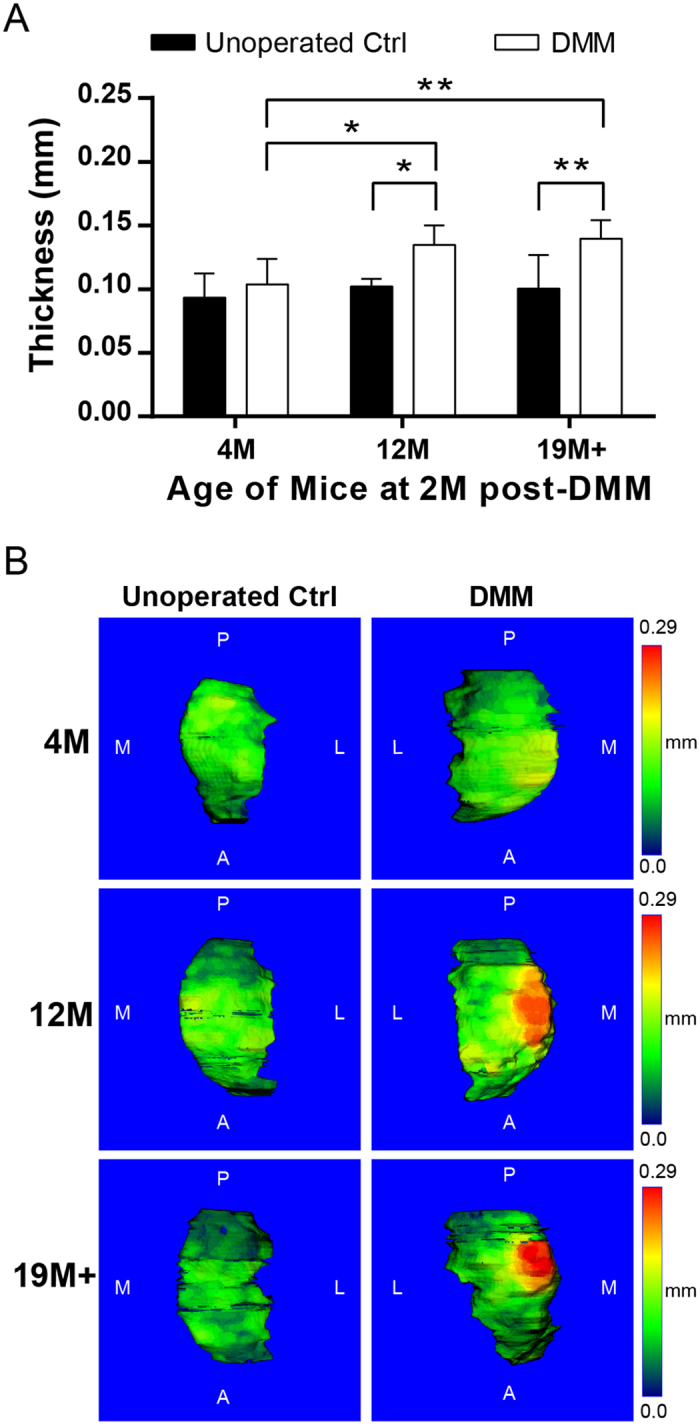
(**A**) Quantification of tibial subchondral bone plate thickness of DMM operated and unoperated contralateral knees of male mice from different age groups. *p-value < 0.05, **p-value < 0.01; (**B**) Representative color maps of subchondral bone plate thickness of DMM operated and unoperated contralateral knees from each age group. Color map legend is scaled to the maximum thickness detected in the 19 M+ group. A = anterior, P = posterior, M = medial, and L = lateral.

The subchondral bone plates of operated knees increased in thickness 2 month after DMM surgery compared to unoperated contralateral knees regardless of age (Fig. 2A). The difference in thickness between the operated and unoperated knee was significant in the 12 M and 19 M+ groups. The 4 M group followed a similar upward trend, but the increase was not statistically significant. In the unoperated control knees, the thickness of the subchondral bone plates was comparable across all age groups. Color maps of the subchondral bone plate thickness confirmed that the 4 M age group exhibited a similar range of thickness between operated and unoperated knees, but in the 12 M and 19 M+ groups, the thickness significantly increased post-DMM (Fig. 2B).

### Bone volume fraction of osteophytes after DMM was significantly higher in older (19 M+) male mice

**Figure 3:**
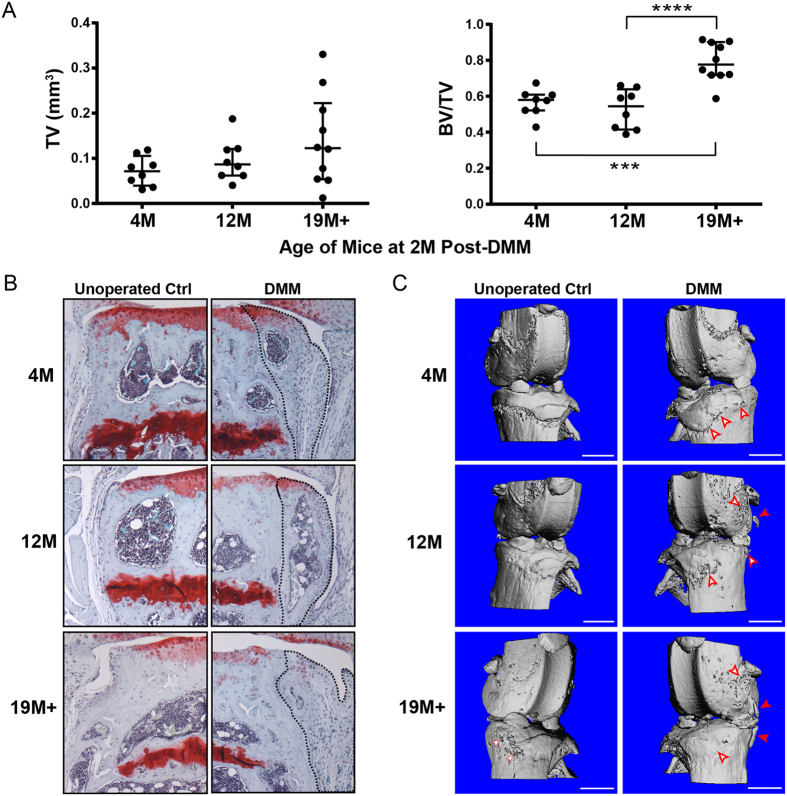
(**A**) Total tissue volume (TV) and bone volume fraction (BV/TV) of contoured osteophytes in DMM operated knees of male mice from different age groups. ***p < 0.001, ****p < 0.0001; (**B**) Representative safranin-O/fast green stained histological sections of the DMM operated knees in male mice from different age groups, with osteophytes contoured by dotted lines; (**C**) Representative 3D reconstructed μCT images revealing distinct surface topographical changes of the knee post-DMM in male mice from different age groups. Open red arrows indicate areas of superficial roughening and/or thickening of bone surface that were not observed in the unoperated knee. Filled red arrows indicate osteophyte protrusions away from subchondral bone surface which were more prominent in the 19 M+ age group. Scale bar = 1 mm.

Osteophytes were detected on all of the DMM operated knees regardless of age, although two knees from the 4 M age group showed only superficial bony outgrowths and were not quantified. None of the unoperated knees showed signs of osteophyte formation, except for one knee in the 19 M+ age group. Quantification of the osteophyte total tissue volume (TV) revealed a trend of increasing size with age, but this was not statistically significant (Fig. 3A, left). The bone volume fraction (BV/TV) of osteophytes was significantly higher in the 19 M+ group compared to the other age groups, while no difference in bone volume fraction was observed between the 4 M and 12 M age groups (Fig. 3A, right). Representative histological images of the osteophyte, as demarcated by a black dashed line, showed a progressive increase in osteophyte size and disappearance of cartilaginous regions and marrow-like cavities with age (Fig. 3B). 3D reconstruction of the μCT images allowed visualization of the bone surface topographical changes between DMM operated and unoperated knees (Fig. 3C). The 4 M unoperated tibia had an even textured surface and a distinct ossification groove that marks the perichondrial ring of the growth plate. In contrast, the 4 M DMM operated knee showed extensive superficial bone thickening that obscured the ossification groove. A few roughened patches (indicated by unfilled arrows) were also observed in the distal femur. In the 12 M group, the ossification groove was less visible in the unoperated tibia than the younger mice while the DMM operated tibia showed extensive bone thickening, prominent rough patches (especially in the distal femur, indicated by unfilled arrows) and appearance of distinct bone projections (indicated by filled arrows). In the 19 M+ group, signs of rough patches were observed both before and after DMM while far more pronounced osteophyte projections were observed post DMM.

### Female mice developed milder OA post-DMM compared to males in the 12 M age group

**Figure 4:**
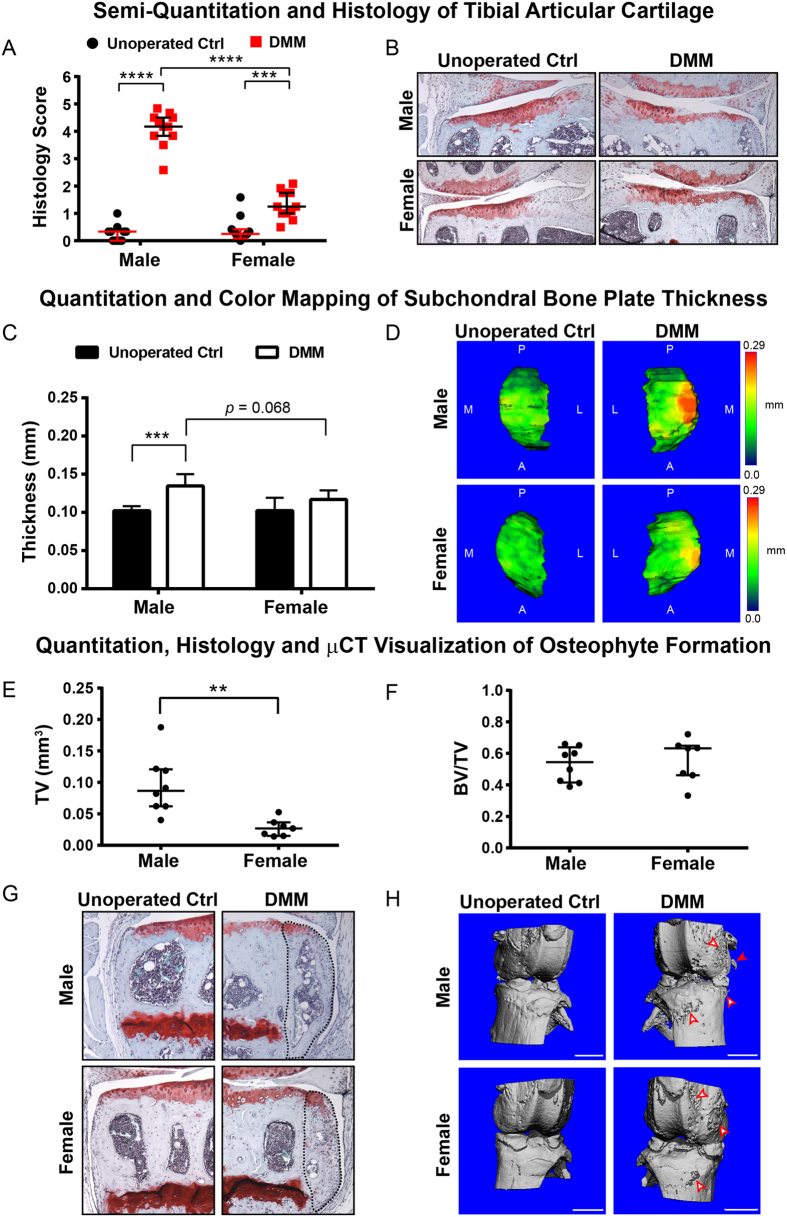
(**A**) Histological score of the tibial articular cartilage from unoperated and DMM operated knees of male vs. female mice from the 12 M age group. Individual scores are plotted along with median score and interquartile range. ***p-value < 0.001, ****p-value < 0.0001; (**B**) Representative safranin-O/fast green stained histological sections of the articular cartilage of unoperated and DMM operated knees of male vs. female mice from the 12 M age group; (**C**) Quantification of tibial subchondral bone plate thickness of DMM operated and unoperated contralateral knees of male vs. female mice from the 12 M age group. **p-value < 0.01; (**D**) Representative color maps of subchondral bone plate thickness of DMM operated and unoperated contralateral knee of male vs. female mice from the 12 M age group. Color map legend is scaled to the maximum thickness detected in the 19 M+ age group. A = anterior, P = posterior, M = medial, and L = lateral; (**E**) Total tissue volume (TV) and (**F**) bone volume fraction (BV/TV) of contoured osteophytes in DMM operated knees from male vs. female mice from the 12 M age group. **p < 0.01; (**G**) Representative safranin-O/fast green stained histological sections showing the presence of osteophyte (dashed line) in the DMM operated knees in male vs. female mice from the 12 M age group; (H) Representative 3D reconstructed μCT images showing distinct surface topographical changes of the knee post-DMM in male vs. female mice from the 12 M age group. Open red arrows indicate superficial roughening and/or thickening of bone surface. Filled red arrows indicate osteophyte protrusions away from subchondral bone surface. Scale bar = 1 mm.

Female mice from the 12 M group developed milder OA while their male counterparts developed more severe OA post-DMM (Fig. 4A). The median histological score of the articular cartilage from unoperated female knees was 0.25 (0.08–0.42) compared to a score of 0.33 (0.00–0.33) in the males, reflecting normal cartilage structures for both genders (Fig. 4B). In the DMM operated female knees, the median histological score was 1.3 (1.0–1.8) compared to the operated male knees of 4.2 (3.8–4.5). Histological images showed that the articular cartilage damage was primarily limited to superficial fibrillations and loss of safranin-O/fast green staining in the 12 M female post-DMM, while the male operated knees showed more severe cartilage erosions down to the calcified cartilage (Fig. 4B). The degrees of subchondral bone sclerosis between 12 M male and female DMM operated knees were also very different despite similar bone plate thickness in the unoperated knees. No statistically significant increase in subchondral bone plate thickness was observed in the females while significant thickening was observed in the males after DMM (Fig. 4C). Color maps of bone thickness showed a distinct localization of the thickened region in the subchondral bone plate of the male operated knee while the spatial variation of subchondral bone plate thickness between unoperated and operated knees remained similar in the 12 M female (Fig. 4D). Finally, smaller osteophytes were detected in 12 M female DMM operated knees (TV = 0.028 ± 0.014 mm^3^) compared to males (TV = 0.096 ± 0.047 mm^3^) (Fig. 4E), but the bone volume fraction of the osteophytes were comparable between the two genders (Fig. 4F). Histology also confirmed smaller osteophytes in the female DMM knees, which were also characterized by the presence of marrow-like cavities (Fig. 4G). 3D reconstruction of the μCT images detected visible projections of osteophytes extending out from the subchondral bone (Fig. 4H, indicated by filled arrows) in DMM operated males but not in the females.

### With advanced age, female mice showed signs of severe OA and subchondral bone changes post-DMM

Of the female mice at 21 M (n = 4) that underwent DMM surgery, two showed signs of severe OA cartilage with scores of 5.0 and 6.0 while the other two had scores of 1.2 and 2.2, which were comparable to the scores from the female 12 M group post-DMM (Supplementary Figure 4A,B). The difference in subchondral bone plate thickness with and without DMM surgery was also significant for female mice in the 21 M (n = 3) group but not in the 12 M (n = 7) or 18 M (n = 3) groups (Supplementary Figure 4C). Color mapping showed that the overall subchondral bone changes with and without DMM were very mild compared to male counterparts (Supplementary Figure 4D) and that the difference observed in the 21 M group was a result of severe osteoporosis in the unoperated control (Supplementary Figure 4E). The sizes of the osteophyte detected in the DMM-operated knees were comparable across all female age groups (Supplementary Figure 4F) but the BV/TV of the osteophytes was highest post-DMM in the 18 M group (Supplementary Figure 4G).

## Discussion

Multiple tissue compartments within the knee undergo significant structural changes during OA development. Using C57BL/6 mice from 3 age groups, we determined that articular cartilage and subchondral bone respond to DMM differently with age. The DMM surgery, popularized by Glasson *et al*.^11^, is considered a minimally invasive surgery that mimicked human OA following meniscal injury. However, most murine OA studies that perform DMM do not adequately address age-related perturbations in joint mechanics or cellular processes. Results from this study reaffirm that age is an important factor in dictating how various compartments in the mouse joint respond to DMM-induced OA and should be considered in determining the translatability between murine OA models and human OA.

Subchondral bone plate sclerosis and articular cartilage degeneration are both hallmarks of mouse^18^ and human OA^27^. We showed that in male mice, the thickening of DMM-induced subchondral bone plate accompanied increased severity of degradation in the overlying cartilage. However, between 12 M and 19 M+ groups, we did not observe significant differences between the degrees of cartilage degradation and subchondral bone thickening. Interestingly, although the 12 M and 19 M+ groups both weighed significantly more than the 4 M group, no significant further increase in body weight from 12 M to 19 M+ was detected (Supplementary Information Fig. 1A). Whether the observed age-related changes can be attributed to altered joint mechanics (driven by weight gain or reduced physical activity) or by age-related cellular metabolism and function requires additional studies. Given the need for more reproducible murine models to study OA^28^, our results suggest that performing DMM surgery on middle-aged male mice (12 M group) could be advantageous in producing more uniform and reproducible OA phenotypes than in younger mice (2 M group). The 19 M+ group also developed more severe cartilage degradation post-DMM than the 4 M group, but the histology scores were not as uniform as the 12 M group. Possible reasons include the wider age range (19 to 22 months at time of analysis) within this group and other unexplored comorbidities that accompany advanced age.

Osteophyte formation was detected in all DMM operated knees regardless of age, but the size and shape varied. Among the three age groups, the size of the osteophyte post-DMM trended up with age but the increase was not significant (Fig. 3A, *left*). The size^29^ and direction^30^ of the osteophyte have been associated with the degree of cartilage erosion, but this correlation was not evident from our data. No significant differences in the size or bone volume fraction of osteophytes was observed between 4 M and 12 M age groups where the greatest difference in cartilage and subchondral bone plate changes were detected. The direction of the bony outgrowths were also confined to the horizontal axis and never in the vertical axis, which is common in advanced human OA. Overall, our data with aged male mice do not support a strong correlation between the extent of osteophyte formation/remodeling and the degrees of articular cartilage erosion or subchondral bone plate thickening. This finding is in line with previous observations that osteophytes can develop in the absence of overt cartilage damage^6^,^31^ and in some animal models is visible a few days after joint injury^32^. Nevertheless, it is still possible that cartilage and subchondral bone plate can still affect osteophyte development through soluble factors. Interestingly, we found that bone volume fraction of the osteophytes in the 19 M+ age group was significantly higher than those in the younger age groups, consistent with more extensive bone remodeling/faster bone turnover reported within more mature osteophytes^33^,^34^. The appearance of central ossification coupled with fewer cartilaginous regions and marrow-like cavities in the 19 M+ osteophyte was also consistent with more extensively remolded bone found in later stages of osteophyte development^34^. While higher bone turnover in the subchondral bone has been associated with advanced stages of human OA^35^,^36^,^37^, further studies are necessary to understand the relationship among different stages of osteophytes and OA progression.

Sex hormones play a significant role in OA severity in the murine DMM model, where young 4.5 M males develop more severe OA than age-matched females^26^. Given the more consistent OA phenotype in 12 M males, we examined whether gender differences in DMM-induced OA severity persisted at this age. We found that at 12 M, female mice continue to develop milder OA compared to age-matched males, characterized by less articular cartilage degradation, less subchondral bone sclerosis, and smaller osteophytes. This milder OA in the 12 M females also reflects the same association between cartilage erosion and subchondral bone plate thickening found in males. Since C57BL/6 female mice produce litters up to 15 months of age^38^, the amount of circulating estrogen in 12 M females may be sufficient to still protect against more severe OA in spite of their increasing osteoporosis risks and weight gains^20^. It should be noted, however, the weight factor cannot be ruled out from the observed gender-specific differences as 12 M female mice weighed significantly less than 12 M male counterparts (Supplementary Information Fig. 1B).

Although not the focus of this study, examination of the contralateral unoperated joint controls revealed that spontaneous OA due to age was very mild in nature. The age-induced OA changes were characterized with mainly progressive loss of safranin-O staining of the articular cartilage but no significant structural perturbations (superficial fibrillations only observed in some of the 19 M+ group). We also did not observe significant changes in subchondral bone with age except for the very old (21 M) females where a reduction in subchondral bone mineral density consistent with osteoporosis was observed.

There are several limitations in this study. First, we validated the efficacy of our DMM procedure with sham operation controls (opening and closing of skin and joint capsule) in the 2 M aged groups prior to the study where OA was only found in DMM-operated but not sham-operated joints (comparable to non-operated control), but such validation was not performed in the other older age groups. It should be noted, however, that the limited literature^26^ on relatively old mice demonstrated no difference between sham and unoperated control in 12 M male mice. In addition to articular cartilage and subchondral bone, synovial inflammation also plays a role in OA development^39^ and is also age-dependent^25^. Detecting markers of inflammation in the synovial fluid or in the serum could provide insight into age-related difference in the degree or onset of joint inflammation. Finally, the observation that females were still protected from DMM-induced OA at 12 M begs the questions as to whether such protection wears off in even older females. Since this was not the initial focus of our study, we used only a limited number of aged female mice to preliminarily evaluate the structural joint changes in much older (18 M and 21 M) females. We observed severe cartilage erosions in some 21 M females while others showed only mild cartilage erosions similar to those seen in the 12 M age group. This suggests that the protective effect of sex hormone may have started to wear off at 21 M. It was only in the 21 M female group that we observed significant differences in subchondral bone plate thickness between DMM operated and unoeprated knees which we attribute to the severe thinning of the subchondral bone prior to DMM due to osteoporosis. For future comprehensive studies investigating the role of sex hormone in OA development in old female mice, it would be valuable to extend the examination beyond 2-month post-DMM as others have shown that sex hormone protection against DMM-induced OA in younger females could wear off after 12 weeks^40^.

In conclusion, we showed that the increasing severity of DMM-induced cartilage degradation as a function of age was accompanied with more pronounced subchondral bone plate thickening and to a lesser extent increasing size of osteophytes. Our findings support previous work^41^ that utilizes non-invasive monitoring of subchondral bone changes as an accurate method for evaluating OA severity longitudinally. Future work combining contrast-enabled μCT quantitation^42^ of cartilage changes with subchondral bone plate thickness changes could help establish better temporal correlations between these two structures during OA development. Our study supports the view^43^ that age is an important factor in dictating structural changes (accelerated cartilage loss coupled with subchondral bone plate sclerosis and enhanced osteophyte maturation) post-DMM. Accordingly, we suggest that therapeutic effects of potential disease modifying OA targets observed in young adult mice using DMM-induced OA should also be validated in older mice. Our data also suggest that DMM-induced OA in older 12 M mice is more reproducible and may be more relevant than 4 M mice for identifying and translating potential disease modifying OA targets to human therapy.

## Methods

### Animals

Wild-type C57BL/6 mice were used in this study. The mice were divided into three age groups corresponding to 4 month old (4 M, male), 12 month old (12 M, male and female) and 18–22 month old (19 M+, male; 18 M and 21 M, female) mice at 2 months post-DMM surgery. All mice were housed in a fully accredited Animal Care facility. All procedures and experiments were approved by the University of Massachusetts Medical School Institutional Animal Care and Use Committee (IACUC), and performed in accordance with the relevant guidelines, regulations and approved IACUC protocol.

### DMM Surgery

Surgery was performed on the right knee of mice at 2 months (male, n = 13), 10 months (male, n = 11; female, n = 11), 17–20 months (male, n = 11), and 19 months (females, n = 4) as previously described^11^,^19^. Briefly, a 3–4 millimeter cut was made in the skin to expose the joint capsule. A medial parapatellar incision was made to open the joint capsule and expose the medial meniscus. After transection of the meniscotibial ligament, the joint capsule and skin were closed using 7–0 sutures. The contralateral knee was not operated and served as a control. Mice were allowed to freely move in the cage immediately after surgery. Analgesic and antibiotic were administered for 2 days post-surgery per IACUC protocol.

### Histology Analysis

Histological scoring criterion (Supplementary Table 1) for articular cartilage was based on previous literature^44^ and detailed in our recent study^19^. In this study, we focused our analysis on the medial tibial plateau since it is known to be more susceptible to damage after DMM^18^ and we confirmed that it is indeed the case even in the 19 M+ age group. Furthermore, we avoided femoral condyle analysis as we found that it revealed less consistent results with changes in cartilage and the subchondral bone due to its greater degree of rotation. The operated and unoperated knees were harvested and fixed in an extended position using 10% neutral buffered formalin and decalcified in 20% EDTA for 14 days. Specimens were paraffin embedded, cut into 5-μm sections, and stained with safranin-O/fast green. The medial tibial plateau from six equally-spaced sections were blindly scored by three individuals and averaged. For each age group, the individual and median scores for the unoperated and operated knees were plotted along with the interquartile range.

### μCT Analysis

Mice knees from each age group (4 M male, n = 9; 12 M male, n = 8; 12 M female, n = 7; 18 M female, n = 3; 19 M+ male, n = 7; 21 M female, n = 3) were scanned on a μCT 40 scanner (Scanco Medical, Brüttisellen, Switzerland) at a 10-μm voxel resolution. To determine subchondral bone plate thickness, the cortical bone of the medial tibial plateau was contoured to exclude the calcified articular cartilage and any portion that is part of an osteophyte (Supplementary Figure 2A). The thickness was calculated using Scanco’s trabecular bone evaluation (Direct method). Color maps of the thickness were generated from the contoured subchondral bone plate of the operated and unoperated knees. To better visualize the thickened region post-DMM, the color legends were scaled to the maximum thickness detected in each age group (Supplementary Figure 3). Only well-demarcated osteophytes that projected outward from the subchondral bone of DMM operated knees were contoured (Supplementary Figure 2B) and quantified for total osteophyte tissue volume (TV) and bone volume fraction (BV/TV). 3D reconstruction of the knees was carried out to visualize topographical changes on the bone surface as well as the extent of bony outgrowths as a function of age, gender and DMM. Quantitative μCT data were presented as mean ± standard deviation.

### Statistical Analysis

All statistical analysis was performed using GraphPad Prism version 7.0 (La Jolla, CA). Analysis of the histological scores was performed using Mann-Whitney non-parameteric *t*-test. Analysis of all μCT data was performed using 2-way ANOVA and Tukey’s multiple comparisons test. *P*-value < 0.05 was considered significant.

## Additional Information

**How to cite this article:** Huang, H. *et al*. Age-dependent Changes in the Articular Cartilage and Subchondral Bone of C57BL/6 Mice after Surgical Destabilization of Medial Meniscus. *Sci. Rep.* **7**, 42294; doi: 10.1038/srep42294 (2017).

**Publisher's note:** Springer Nature remains neutral with regard to jurisdictional claims in published maps and institutional affiliations.

## Supplementary Material

Supplementary Information
