# Supplementary material for: Age-dependent Changes in the Articular Cartilage and Subchondral Bone of C57BL/6 Mice after Surgical Destabilization of Medial Meniscus
Source: Sci Rep. 2017 Feb 9;7:42294. doi: 10.1038/srep42294 (PMC5299455; doi:10.1038/srep42294)
Supplement: Supplementary Information [file srep42294-s1.pdf]

Supplementary Information

Age-dependent Changes in the Articular Cartilage and Subchondral Bone of C57BL/6 Mice after Surgical Destabilization of Medial Meniscus

Henry Huang, Jordan D. Skelly, David C. Ayers and Jie Song

Department of Orthopedics & Physical Rehabilitation, University of Massachusetts Medical School, Worcester, MA 01655, USA

Grade	OA Severity	Description	Representative Image
0	Normal	Normal	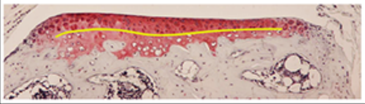
0.5	Mild	Loss of Safranin-O staining without structural change	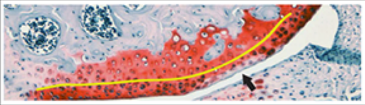
1	Mild	Roughened articular surface and small fibrillations	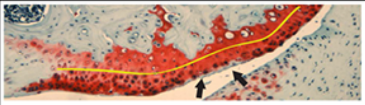
2	Moderate	Fibrillations or vertical cleft extending below superficial layer	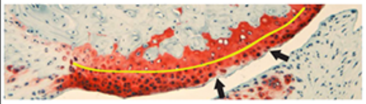
3	Moderate	Vertical clefts/erosions extending to calcified layer with <25% of articular surface affected.	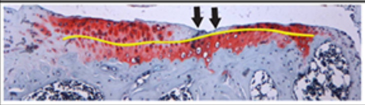
4	Severe	Vertical clefts/erosions extending to calcified layer with 25%-50% of articular surface affected.	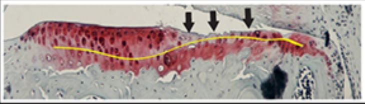
5	Very Severe	Vertical clefts/erosions extending to calcified layer with 50-75% of articular surface affected.	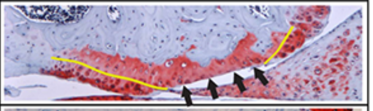
6	Very Severe	Vertical clefts/erosions extending to calcified layer with >75% of articular surface affected.	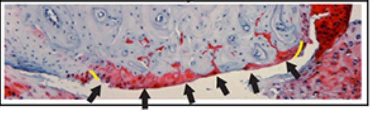

Supplementary Table 1. Articular cartilage histology scoring criterion with representative safranin O-stained cartilage sections. Yellow line indicates tidemark; Black arrows denote loss of staining, fibrillations, or erosions. This table is reprinted from our previous publication "Huang,

H., Veien, E.S., Zhang, H., Ayers, D.C. & Song J. Skeletal Characterization of Smurf2-Deficient Mice and In Vitro Analysis of Smurf2-Deficient Chondrocytes. *PLoS One*. **11,** e0148088; 10.1371/journal.pone.0148088 (2016).” with no modifications and is licensed under CC BY 4.0 (https://creativecommons.org/licenses/by/4.0/)

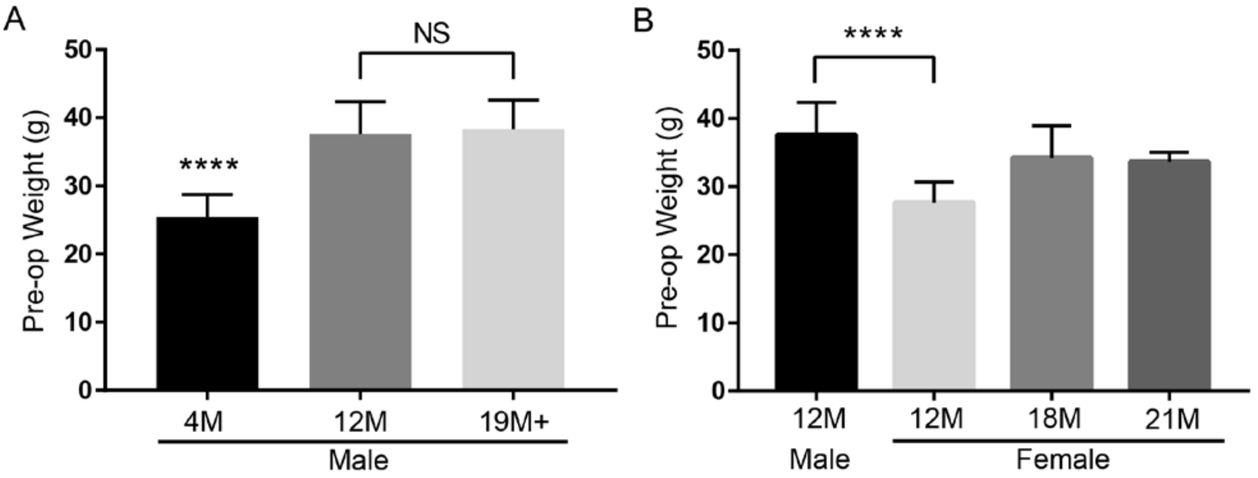

Supplementary Figure 1. (A) Average weight ± SD of male mice prior to DMM surgery in each age group. (B) Average weight ± SD of 12M male compared to female mice of different ages prior to DMM surgery. ****$p < 0.0001$

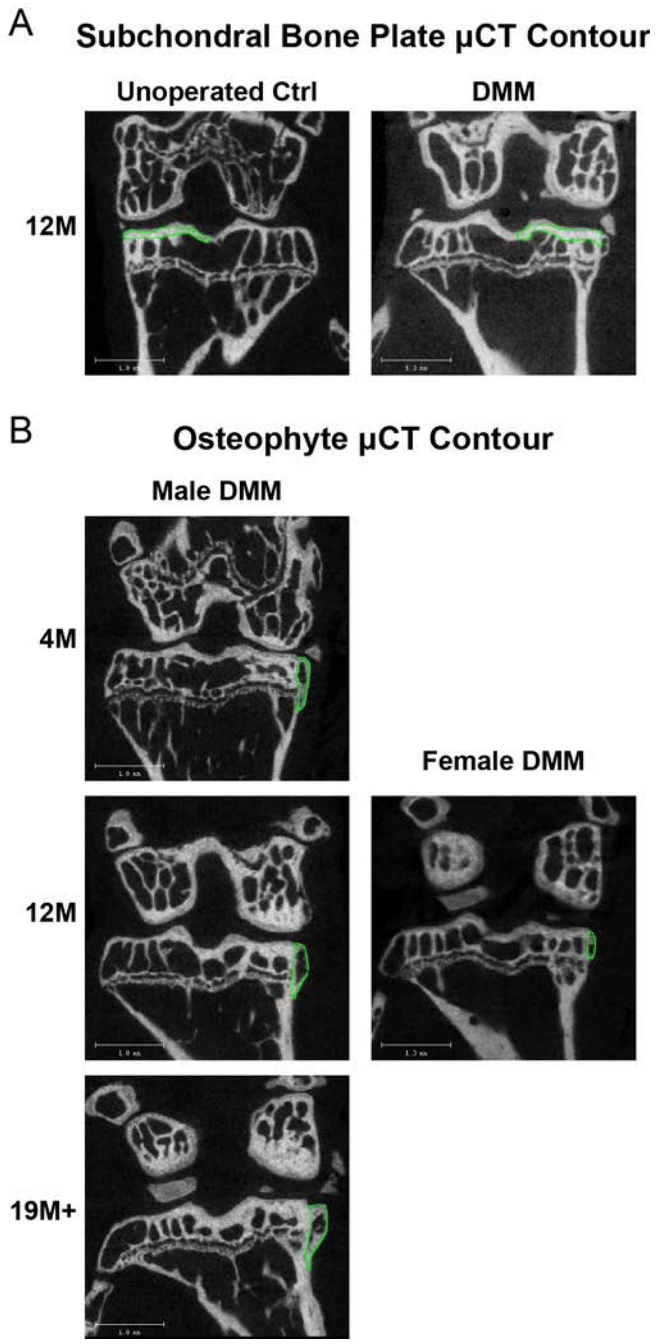

Supplementary Figure 2. (A) Contouring of tibial subchondral bone plate for the thickness measurement as indicated by green lines; (B) Contouring of osteophyte outgrowth from DMM operated knees of male mice from the three age groups and of females from 12M age group as indicated by green lines.

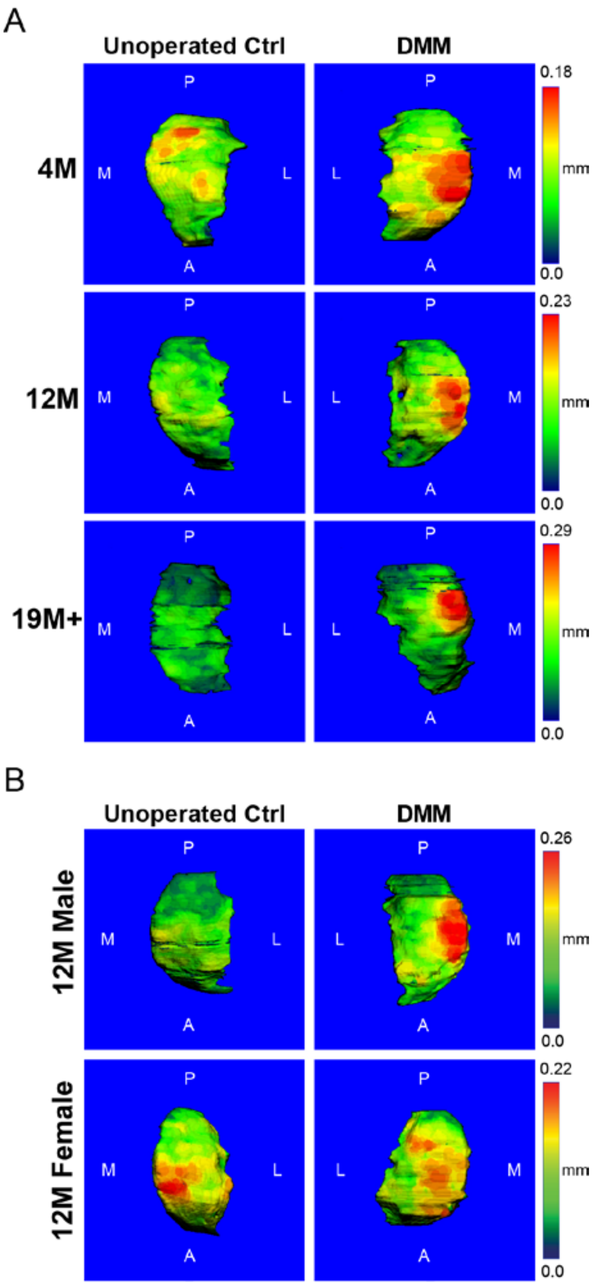

Supplementary Figure 3. (A) Representative color maps of subchondral bone plate thickness of DMM operated and unoperated contralateral knees from each age group; (B) Representative color maps of subchondral bone plate thickness of DMM operated and unoperated contralateral knee of male vs. female mice from the 12M age group. Color map legend is scaled to the maximum thickness detected within each age and gender group. A = anterior, P = posterior, M = medial, and L = lateral.

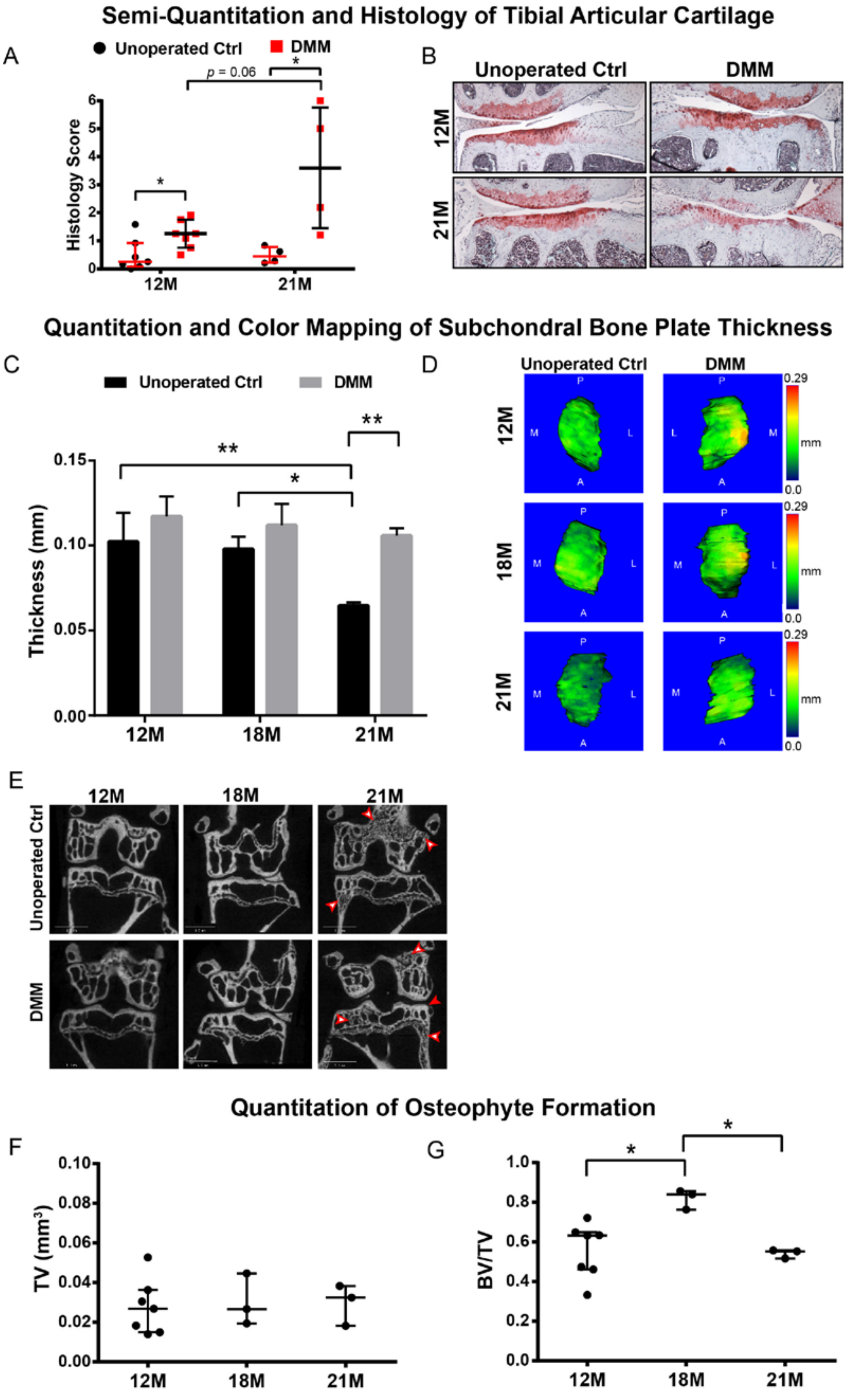

Semi-Quantitation and Histology of Tibial Articular Cartilage
A
Unoperated Ctrl
DMM
p = 0.06
*
Histology Score
12M
21M
B
Unoperated Ctrl
DMM
12M
21M
Quantitation and Color Mapping of Subchondral Bone Plate Thickness
C
Unoperated Ctrl
DMM
**
*
Thickness (mm)
0.15
0.10
0.05
0.00
12M
18M
21M
D
Unoperated Ctrl
DMM
12M
18M
21M
P
A
M
L
0.29
mm
0.0
E
12M
18M
21M
Unoperated Ctrl
DMM
Quantitation of Osteophyte Formation
F
TV (mm³)
0.10
0.08
0.06
0.04
0.02
0.00
12M
18M
21M
G
BV/TV
1.0
0.8
0.6
0.4
0.2
0.0
*
12M
18M
21M

Supplementary Figure 4. (A) Histological scores and (B) representative safranin-O/fast green stained histological sections of the tibial articular cartilage from unoperated and DMM operated knees of female mice from the 12M and 21M age groups. Individual scores are plotted along with median score and interquartile range. $*p < 0.05$; (C) Quantification and (D) representative color maps of tibial subchondral bone plate thickness of DMM operated and unoperated contralateral knees of female mice from the 12M, 18M and 21M age groups. * p-value < 0.05, **p-value < 0.01; Color map legend is scaled to the maximum thickness detected in the male 19M+ age group. A = anterior, P = posterior, M = medial, and L = lateral; (E) μCT images of longitudinal cross sections of DMM operated and unoperated contraleral knees of female mice from 12M, 18M and 21M. Open arrows indicate areas of osteoporosis and closed arrow indicates region of enhanced bone remodeling after DMM surgery. (F) Total tissue volume (TV) and (G) bone volume fraction (BV/TV) of contoured osteophytes in DMM operated knees of female mice from the 12M, 18M and 21M age group. $*p < 0.05$.
